# An Association of an eBURST Group With Triazole Resistance of *Candida tropicalis* Blood Isolates

**DOI:** 10.3389/fmicb.2020.00934

**Published:** 2020-05-19

**Authors:** Orawan Tulyaprawat, Sujiraphong Pharkjaksu, Piriyaporn Chongtrakool, Popchai Ngamskulrungroj

**Affiliations:** Department of Microbiology, Faculty of Medicine Siriraj Hospital, Mahidol University, Bangkok, Thailand

**Keywords:** *Candida tropicalis*, resistance, Thailand, candidemia, prevalence, virulence factor, MLST, prevalence shift

## Abstract

Candidemia, a bloodstream infection caused by genus *Candida*, has a high mortality rate. *Candida albicans* was previously reported to be the most common causative species among candidemia patients. However, during the past 10 years in Thailand, *Candida tropicalis* has been recovered from blood more frequently than *C. albicans*. The cause of this shift in the prevalence of *Candida* spp. remains unexplored. We conducted *in vitro* virulence studies and antifungal susceptibility profiles of 48 *C. tropicalis* blood isolates collected during 2015–2017. To compare to global isolates of *C. tropicalis*, multilocus sequence typing (MLST), a minimum spanning tree, and an eBURST analysis were also conducted. *C. tropicalis* and *C. albicans* were the most (47–48.7%) and second-most (21.5–33.9%) common species to be isolated from candidemia patients, respectively. Of the *C. tropicalis* blood isolates, 29.2, 0, 100, and 93.8% exhibited proteinase activity, phospholipase activity, hemolytic activity, and biofilm formation, respectively. Moreover, 20.8% (10/48) of the isolates were resistant to voriconazole and fluconazole, and also showed high minimum inhibitory concentrations (MICs) to posaconazole and itraconazole. In contrast, most of the isolates were susceptible to anidulafungin (97.9%), micafungin (97.9%), and caspofungin (97.9%), and showed low MICs to amphotericin B (100%) and 5-flucytosine (100%). The MLST identified 22 diploid sequence types. Based on the eBURST analysis and minimum spanning tree, 9 out of 13 members (69.2%) of an eBURST group 3 were resistant to voriconazole and fluconazole, and also showed high MICs to posaconazole and itraconazole. Association analysis revealed the eBURST group 3 was significantly associated with the four triazole resistance (*p* < 0.001). In conclusion, the eBURST group 3 was associated with the triazole resistance and resistance to many antifungal drugs might be collectively responsible for the prevalence shift.

## Introduction

*Candida* species is a cause of mild superficial to serious invasive infections in humans worldwide. The invasive infection often leads to significant morbidity and mortality, particularly with immunocompromised patients (Brown et al., 2012). *Candida* spp. was ranked as the fourth most frequent cause of bloodstream infections with a high mortality rate (Wisplinghoff et al., 2004). Several predisposing factors have been identified for candidemia in hospitalized patients, including parental nutrition, central venous catheterization, organ transplantation, usage of broad-spectrum antibiotics, and longer intensive care unit hospitalization (Kibbler, 1996; Conde-Rosa et al., 2010; Yapar, 2014).

Previously, *Candida albicans* was globally reported to be the most common causative species isolated from the blood of candidemia patients (Guinea, 2014). A large surveillance study in 1997–2016 reported that 46.3–57.4% of all candidemia cases were caused by *C. albicans* whereas only 8.3–10.7% were caused by *Candida tropicalis* (Pfaller et al., 2019). Although considered to be a less prevalent candidemia-causing species, bloodstream infections due to *C. tropicalis* are continuing to rise globally (Guinea, 2014). In the Asian-Pacific and Latin American regions, *C. tropicalis* has been ranked as the first and second most prevalent pathogenic *Candida* species, respectively (Tan et al., 2015; Doi et al., 2016). The mechanism for the increasing prevalence of *C. tropicalis* remains unclear. However, one report revealed that *C. tropicalis* has a higher rate of fluconazole resistance than *C. albicans* (Khairat et al., 2019). An investigation in Southern India, *C. tropicalis* a predominant species (54.3%), also demonstrated that the ability to develop rapid resistance to fluconazole involves the increasing prevalence (YeSudhaSon and MohanraM, 2015).

Resistance to antifungal agents and strong virulence phenotypes of *C. tropicalis* have been reported (Kothavade et al., 2010; Deorukhkar et al., 2014; Fan et al., 2017). Although *Candida* spp. is generally susceptible to most antifungal drugs, the SENTRY Antimicrobial Surveillance Program reported a high fluconazole resistance rate (9.2%) among *C. tropicalis* isolates in Asia-Pacific (Pfaller et al., 2019). Remarkable increasing trends of triazole resistance have also been found in China and Taiwan (Chou et al., 2007; Fan et al., 2017). As a human pathogen, *C. tropicalis* secretes hydrolytic enzymes, namely, proteinase and phospholipase, that digest the host cell membrane, resist phagocytosis, and invade tissues (Naglik et al., 2003). In fact, one study showed a strong proteinase and phospholipase production by *C. tropicalis* isolated from various clinical specimens (Deorukhkar et al., 2014). Hemolysin is another group of enzymes in which *C. tropicalis* can secrete to lyse human red blood cells. The hemoglobin released from the lysed red blood cells is later used as an iron source to facilitate hyphal penetration and yeast dissemination (Furlaneto et al., 2015). A previous report demonstrated that all *C. tropicalis* isolated from the bloodstream and urinary tract infections were able to express hemolytic activity (Negri et al., 2010).

Biofilm formation is also found to play a major role in *Candida* pathogenesis by protecting the pathogenic yeast from the host immune cells and causing resistance to antifungal treatment. Moreover, the biofilm formation also facilitates *Candida* adherence to indwelling medical devices such as vascular catheters, artificial joints, and cardiac devices (Cavalheiro and Teixeira, 2018). Evidence of *C. tropicalis* biofilm formation was provided by a Spain study in which 72.7% of the *C. tropicalis* isolates from both sterile and non-sterile sites were found to be high biofilm producers (Guembe et al., 2017).

Multilocus sequence typing (MLST) has recently been utilized to analyze the pattern of genetic variation of *C. tropicalis* (Tavanti et al., 2005). To date, 543 allele types (AT) and 914 diploid sequence types (DST) have been deposited in the *C. tropicalis* MLST database^1^. Studies of *C. tropicalis* in Kuwait and China have identified more than 59 DSTs of 63 isolates and 94 DSTs of 116 isolates, respectively (Al-Obaid et al., 2017; Wu et al., 2017). Moreover, associations of MLST genotypes with virulence phenotypes/antimicrobial susceptibility patterns have been reported. For example, a study in China showed a significant association between biofilm formation and MLST groups (Yu et al., 2017). Molecular epidemiology in Taiwan has also found a correlation between an MLST cluster and fluconazole resistance (Chou et al., 2007). Furthermore, the relationship between epidemiological data and microsatellite markers illustrated that an increase of *C. tropicalis* resistance to 5-flucytosine was brought by widespread of a flucytosine-resistant clone among hospitalized patients in Paris (Desnos-Ollivier et al., 2008). However, despite the high burden of *C. tropicalis* infections, information on the molecular epidemiology of *C. tropicalis* in Asia is fairly limited.

As a mechanism of the increased prevalence of *C. tropicalis* in Thailand remains controversial, we investigated the molecular epidemiology of *C. tropicalis* recovered from blood cultures at a tertiary care hospital in Thailand. The virulence characteristics and antifungal susceptibilities were also reported. The relationships between MLST-based genetic clusters and antimicrobial susceptibility testing were also described.

## Materials and Methods

### Isolates Collection

Upon approval by the Siriraj Institutional Review Board (COA number: SI 091/2016), information on all *Candida* isolates from positive hemocultures during 2015–2017 were collected from the Department of Microbiology, Faculty of Medicine Siriraj Hospital, Mahidol University, Bangkok, Thailand. To reduce selection bias, two-three *C. tropicalis* blood isolates per month were randomly collected between August 2015 and May 2017 from a culture collection of the diagnostic microbiology laboratory, Department of Microbiology, Faculty of Medicine, Siriraj Hospital. Finally, a total of viable 48 isolates of the *C. tropicalis* blood isolates was included for a molecular epidemiological study. Species identification of *Candida* was primarily performed by using CHROMagar *Candida* chromogenic media (Oxoid, Basingstoke, United Kingdom) and Remel RapID^TM^ Yeast Plus System (Thermo Fisher Scientific, Waltham, MA, United States). To confirm the species-level identification, internal transcribed spacer (ITS) regions of the 48 studied isolates were amplified and analyzed as previously described (Pharkjaksu et al., 2018).

### Investigation of *in vitro* Virulence Factors

Four virulence factors, comprising phospholipase production, proteinase secretion, hemolysin production, and biofilm formation, were measured in triplicates (Pham et al., 2019). The phospholipase, proteinase, and hemolysin productions were tested by inoculating yeast cells onto an egg yolk medium, a bovine serum albumin (BSA) medium, and a sugar-enriched sheep blood medium, respectively (Sachin et al., 2012). First, the yeast isolates were pregrown on Sabouraud dextrose agar (SDA) at 30°C for 48 h before being suspended in a phosphate buffer to make a yeast suspension of 10^8^ CFU/ml. Five microliters of the suspension was spotted onto the egg yolk medium (1.48% w/v CaCl_2_.2H_2_O, 11.7% w/v NaCl, 13% w/v SDA, and 10% v/v egg yolk), the BSA medium (0.01% w/v yeast extract, 0.2% w/v BSA, 1.17% w/v yeast carbon base, and 2% w/v agar), and the sugar-enriched sheep blood medium (1% w/v peptone, 1% w/v yeast extract, 5% w/v sheep blood, 7% w/v dextrose, and 2% w/v agar), and then incubated at 37°C for 48 h. Production of extracellular phospholipase and proteinase was observed by a precipitation zone around the colony and a clear halo zone surrounding the colony, respectively. Production of proteinase and phospholipase was classified into level by the ratio of colony diameter to the diameter of precipitation or clear zone (Pz) as follows: Pz ≤ 0.69, very strong; 0.70 ≤ Pz < 0.79, strong; 0.80 ≤ Pz < 0.89, medium; 0.90 ≤ Pz < 0.99, weak; and Pz = 1, negative. For the hemolysin production, a distinct translucent halo zone around the colony indicated hemolytic activity. Hemolytic activity was measured based on the ratio of the colony diameter to the diameter of the translucent halo zone (Pz) as follows: Pz < 0.64, strongly positive; 0.64 ≤ Pz < 1.00, positive; and Pz = 1, negative.

Biofilm formation of *C. tropicalis* was measured by an XTT [2,3-bis(2-methoxy-4-nitro-5-sulfophenyl)-2H-tetrazolium-5- carboxanilide] reduction assay (Loures and Levitz, 2015). *C. tropicalis*, pregrown on SDA at 30°C for 48 h, was used to prepare a cell suspension of 10^8^ CFU/ml in a yeast extract peptone dextrose broth (1% w/v yeast extract, 2% w/v peptone, and 1% w/v dextrose). One hundred microliters of the suspension were seeded into each well of a flat-bottomed, 96-well plate and incubated at 37°C for 48 h. Non-adherence cells were removed by washing twice with phosphate buffer. The remaining adhered cells were measured for biofilm formation by adding 50 μl of XTT and phenazine methosulfate mixture. After further incubation for 2 h in the dark, the quantity of the biofilm layer was assessed by absorbance at 490 nm. Finally, biofilm formation was classified as: O.D. value > GM (geometric mean) = high biofilm formation, O.D. value ≤ GM = low biofilm formation, and O.D. value < 0.10 = negative biofilm formation (Li et al., 2003).

### Antifungal Susceptibility Testing

The susceptibility of *C. tropicalis* to nine antifungal drugs (fluconazole, voriconazole, itraconazole, posaconazole, 5-flucytosine, anidulafungin, micafungin, caspofungin, and amphotericin B) was determined by using Sensititre YeastOne YO10 (SYO; Thermo Fisher Scientific, Waltham, MA, United States), a colorimetric microdilution method, according to the manufacturer’s instructions. Briefly, 20 μl of 0.5 McFarland yeast suspension was transferred into 11 ml of YeastOne inoculum broth to obtain a concentration of 1.5–8 × 10^3^ CFU/ml. Then, 100 μl of the inoculum was inoculated into each well of a YeastOne susceptibility plate. The concentrations of each drug ranged as follows: fluconazole, 0.12–256 μg/ml; voriconazole, 0.008–8 μg/ml; itraconazole, 0.015–16 μg/ml; posaconazole, 0.008–8 μg/ml; 5-flucytosine, 0.06–64 μg/ml; anidulafungin, 0.015–8 μg/ml; micafungin, 0.008–8 μg/ml; caspofungin, 0.008–8 μg/ml; and amphotericin B, 0.12–8 μg/ml. After 24 h of incubation at 35°C, the minimum inhibitory concentration (MIC) was determined from the change of the colorimetric growth indicator according to the manufacturer recommendations. *Candida parapsilosis* ATCC 20019 and *Candida krusei* ATCC 6258 were used as quality controls. The results were interpreted following the recommendations of Clinical Laboratory Standards Institute documents M27-S4 (Clinical and Laboratory Standards Institute, 2012): clinical breakpoints (CBPs) for fluconazole, MIC ≤ 2 μg/ml susceptible, MIC = 4 μg/ml susceptible-dose dependent, MIC ≥ 8 μg/ml resistant; voriconazole, MIC ≤ 0.12 μg/ml susceptible, MIC 0.25–0.5 μg/ml susceptible-dose dependent, MIC ≥ 1 μg/ml resistant; and anidulafungin, micafungin, and caspofungin, MIC ≤ 0.25 μg/ml susceptible, MIC 0.5 μg/ml intermediate, MIC ≥ 1 μg/ml resistant. For antifungal agents which no CBPs, epidemiological cutoff values (ECVs) assigned by CLSI document M59 were used: itraconazole = 0.5 μg/ml, posaconazole = 0.12 μg/ml, and amphotericin B = 2 μg/ml. As CLSI CBP and ECV for 5-flucytosine was not available, ECV = 0.5 μg/ml was used according to a previously published report (Cantón et al., 2012). To ensure the SYO result was not compromised, 38 of the 48 isolates (including all 32 azole-resistant isolates and 6 azole-susceptible isolates) were tested for their MIC level to fluconazole and itraconazole by the original CLSI methods. According to a previous report (Cantón et al., 2012), 82.1% of ECVs estimated by the SYO method was equal to or within one two-fold dilution of those reported for the CLSI method. Our SYO result showed that 100% of fluconazole and 97.3% of itraconazole results were equal to or within one two-fold dilution of those reported for the CLSI method. Therefore, we believed our SYO result was not compromised.

### Multilocus Sequence Typing Analysis

*Candida tropicalis* was pregrown on SDA at 30°C for 48 h, after which genomic DNA was extracted by a standard phenol-chloroform procedure (Danesi et al., 2014). Amplifications of six housekeeping genes—*ICL1*, *MDR1*, *SAPT2*, *SAPT4*, *XYR1*, and *ZWF1a*—were performed according to a previously described protocol (Tavanti et al., 2005). Subsequently, the purified PCR products of each gene were used for bidirectional sequencing using forward and reverse primers from Axil Scientific Pte. Ltd., Singapore. The sequencing results were edited by MEGA7 software^2^. With each fragment, the forward and reverse sequence chromatograms were visually examined for a strong overlapping peak to define heterozygosity. Each heterozygous position was transformed into a degenerate nucleotide according to the IUPAC nucleotide code. Six sequence fragments of the housekeeping genes of each isolate were compared with information on the *C. tropicalis* MLST database^3^ to define AT and DST. The new allelic profiles and new allele combinations were submitted to a curator of the database, Hsiu-Jung Lo, to verify and assign new allele numbers and DSTs, respectively.

### Phylogenetic Analysis

The edited nucleotide sequences of six fragments were concatenated into a single sequence by the MEGA7 software. Degenerate nucleotides were pre-modified for analysis as previously described (Tavanti et al., 2005). The phylogenetic analysis of the 48 *C. tropicalis* clinical isolates was conducted by the unweighted pair group method with arithmetic average (UPGMA) using the MEGA7 software. To illustrate the phylogenetic relatedness between our 48 isolates and global isolates deposited in the database (accessed in August 2019), a minimum spanning tree was constructed using BioNumerics software version 7.6.3 (Applied Maths, Austin, TX, United States). Each genotype cluster was identified by using the goeBURST algorithm version 1.2.1^4^. Isolates were grouped when five out of six alleles were identical (Francisco et al., 2009).

### Statistical Analysis

The statistical analysis was performed with PASW Statistic for Windows (SPSS Inc., Chicago, IL, United States). An association between eBURST group, antifungal susceptibility, and *in vitro* virulence phenotypes was determined using a Chi-square test. Likewise, the significance of the difference in *in vitro* virulence expression and antifungal susceptibility between *C. albicans* and *C. tropicalis* was also determined by the Chi-square test. Finally, *post hoc* analysis using nQuery Advisor (Elashoff, 2000) to ensure a sufficient sample size was performed by estimation of a type II error in the association analysis. A sufficient sample size was achieved when power for a two-sided test reached 90% at a significant level of 5%. The *p*-value of the Chi-square test was obtained from the web-based calculator^5^. A *p*-value of <0.05 was considered to be significant.

## Results

### *In vitro* Virulence Phenotypes Among *C. tropicalis* Blood Isolates

Interestingly, none of the isolates could produce phospholipase, and only 29.2% (14/48 isolates) were able to produce proteinase. In contrast, all of the isolates were able to produce hemolysin, and almost all (93.8%) of the isolates could form biofilm. For hemolysin, 79.2% (38/48 isolates) had strong hemolytic activity. A high biofilm formation was found in 54.2% (26/48 isolates) of the isolates (Table 1).

**TABLE 1:** Virulent properties of 48 *C. tropicalis* clinical isolates in this study.

Virulent factors	Number (%) of isolates				
	
	Negative	Weak/low	Medium/positive	Strong/strong positive/high	Very strong
Proteinase activity^*a*^	34 (70.8)	0 (0)	3 (6.3)	11 (22.9)	0 (0)
Phospholipase activity^*a*^	48 (100)	0 (0)	0 (0)	0 (0)	0 (0)
Hemolytic activity^*b*^	0 (0.0)	NA	10 (20.8)	38 (79.2)	NA
Biofilm formation^*c*^	3 (6.2)	19 (39.6)	NA	26 (54.2)	NA

*^*a*^Proteinase and phospholipase activity: negative, weak, medium, strong, very strong. ^*b*^Hemolytic activity: negative, positive, strong positive. ^*c*^Biofilm formation: negative, low, high. NA, not applicable.*

### Antifungal Susceptibility of *C. tropicalis* Blood Isolates

It was found that 43.8, 43.8, 37.5, and 20.8% of the isolates were resistant to voriconazole and fluconazole, and also showed high MICs to posaconazole and itraconazole, respectively. The MIC90 value of fluconazole was extremely high (256 μg/ml). In contrast, none of the isolates showed high MICs to amphotericin B and 5-flucytosine (Table 2). Only a few isolates were resistant to anidulafungin (2.1%), micafungin (2.1%), and caspofungin (2.1%).

**TABLE 2:** Susceptibility results among 48 *C. tropicalis* clinical isolates.

Antifungal drugs	CPBs (μg/ml)			ECVs (μg/ml)	MIC (μg/ml)			Category number (%)		
				
	S	SDD/I	R		MIC50	MIC90	GM	S/WT	SDD/I	R/NWT
Voriconazole	≤0.12	0.25–0.5	≥1	–	0.12	8	0.33	21 (43.8)	6 (12.4)	21 (43.8)
Fluconazole	≤2	4	≥8	–	2	256	4.46	26 (54.1)	1 (2.1)	21 (43.8)
Posaconazole^*a*^	–	–	–	0.5	0.50	1	0.31	30 (62.5)	NA	18 (37.5)
Itraconazole^*a*^	–	–	–	0.12	0.25	1	0.35	38 (79.2)	NA	10 (20.8)
Anidulafungin	≤0.25	0.5	≥1	–	0.06	0.12	0.07	47 (97.9)	0 (0)	1 (2.1)
Micafungin	≤0.25	0.5	≥1	–	0.03	0.03	0.03	47 (97.9)	0 (0)	1 (2.1)
Caspofungin	≤0.25	0.5	≥1	–	0.06	0.12	0.05	47 (97.9)	0 (0)	1 (2.1)
Amphotericin B^*a*^	–	–	–	2	1	1.2	0.95	48 (100.0)	NA	0 (0)
5-Flucytosine^*a*^	–	–	–	0.5	0.12	0.2	0.09	48 (100.0)	NA	0 (0)

*^*a*^Categorization based on the epidemiologic cutoff values (ECVs). CBPs, clinical breakpoints; ECVs, epidemiologic cutoff values; S, susceptible; WT, wild type; SDD, susceptible-dose dependent; I, intermediate; R, resistant; NWT, non-wild type; MIC, minimal inhibitory concentration; GM, geometric mean; NA, not applicable.*

### Azole Drug Resistance Isolates and the UPGMA-Based Cluster

**FIGURE 1:**
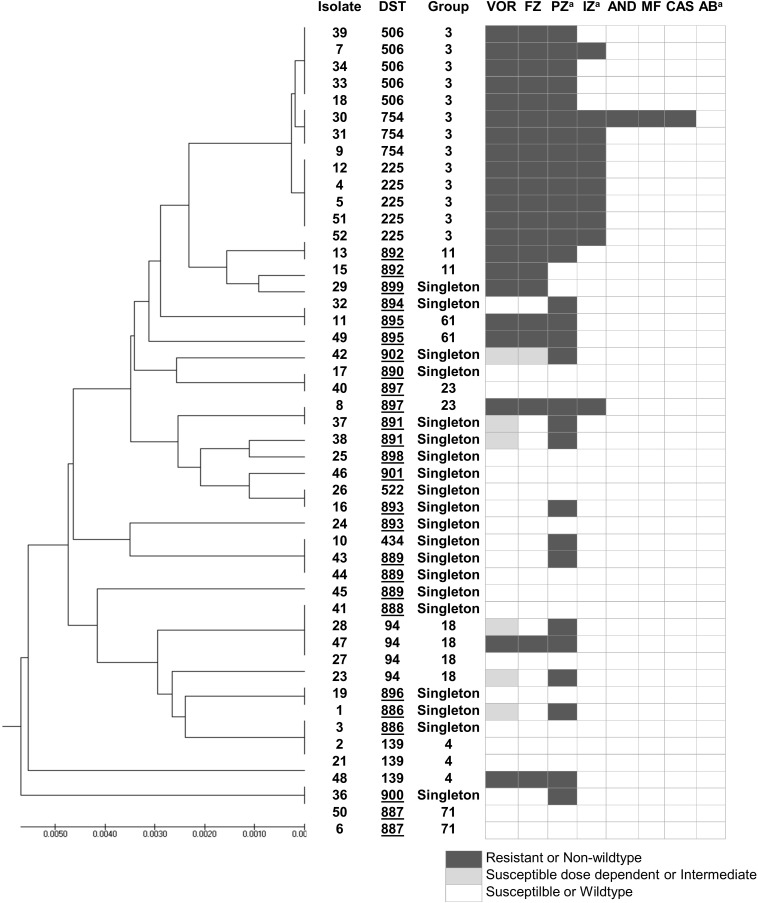
A UPGMA dendrogram based on MLST of six gene fragments against antifungal susceptibility pattern of 48 *C. tropicalis* isolates causing candidemia in Thailand. ^*a*^Susceptibility categorized based on epidemiologic cutoff values; underlined DST numbers, new DST identified in this study; Group, group defined by goeBURST; black box, resistant or non-wild type; gray box, susceptible dose dependent or intermediate; white box, susceptible or wild type. DST, diploid sequence type; VOR, voriconazole; IZ, itraconazole; FZ, fluconazole; AND; anidulafungin; MF, micafungin; CAS, caspofungin; AB, amphotericin B; PZ, posaconazole.

**FIGURE 2:**
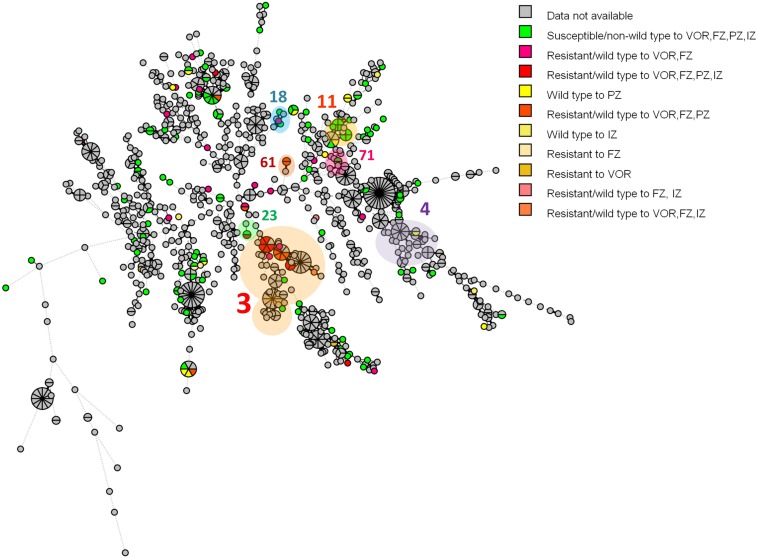
A minimum spanning tree illustrating the relationship between the 48 *C. tropicalis* isolates from Thailand and 1,019 isolates from other countries available from the *C. tropicalis* MLST database as of August 2019. Each circle corresponds to a unique DST; the number outside the circle indicates an eBURST cluster; the size of the circle represents the number of isolates belonging to the same DST; and the colors inside the circle represent voriconazole, fluconazole, posaconazole, and itraconazole susceptibility. VOR, voriconazole; FZ, fluconazole; PZ, posaconazole; IZ, itraconazole.

According to the *C. tropicalis* MLST database, a total of 24 DSTs were identified. Seven DSTs (DST94, DST139, DST225, DST434, DST506, DST522, and DST754; 22 isolates) were already present in the database, while 17 DSTs (26 isolates) were newly identified in this study (Figure 1). Based on the UPGMA dendrogram, most isolates (69.2%, 9/13 isolates) with resistance to voriconazole and fluconazole, and high MICs to posaconazole and itraconazole were on the same cluster (Figure 1). Further analysis with the goeBURST program showed that the azole-resistance cluster belonged to the eBURST group 3 with a high resistance/non-wild type rate of 69.2% (9/13 isolates) while the low rate of 2.9% (1/35 isolates) was found in the non-eBURST group 3 isolates (Table 3 and Figure 1). Other eBURST groups and singletons are illustrated in the Figures 1, 2. The statistical analysis also revealed a significant association between the eBURST group 3 isolates and the four drugs resistance (Table 3; *p* < 0.001). The *post hoc* analysis indicated that the 48 studied isolates of *C. tropicalis* were sufficient for demonstrating the association between the four triazole resistance and eBURST cluster 3. In contrast, there was no significant correlation between the eBURST group 3 isolates with any virulence phenotypes. Other studies also reported that Asia was the origin of most eBURST group 3 isolates (Supplementary Figure S1).

**TABLE 3:** Number of isolates in cluster 3 and non-cluster 3 in correlation to four azole drugs (voriconazole, fluconazole, posaconazole, and itraconazole) susceptibility and four virulence factors.

Drug susceptibility/virulence factors		Number (%) of isolates		*p*-value
		
		Cluster 3	Non-cluster 3	
VOR, FZ, PZ, and IT susceptibility	Resistant/NWT to all four drugs	9 (69.2)	1(2.9)	<0.001
	Not resistant/WT to all four drugs	4 (30.8)	34 (97.1)	
	Total	13 (100.0)	35 (100.0)	

Proteinase activity	Very strong	0 (0.0)	3 (8.6)	0.656
	Strong	3 (23.1)	5 (14.3)	
	Medium	1 (7.7)	2 (5.7)	
	Negative	9 (69.2)	25 (71.4)	
	Total	13 (100.0)	35 (100.0)	

Hemolytic activity	Strong positive	9 (69.2)	29 (82.9)	0.302
	Positive	4 (30.8)	6 (17.1)	
	Negative	0 (0.0)	0 (0.0)	
	Total	13 (100.0)	35 (100.0)	

Biofilm formation	High	9 (69.2)	17 (48.6)	0.362
	Low	3 (23.1)	16 (45.7)	
	Negative	1 (7.7)	2 (5.7)	
	Total	13 (100.0)	35 (100.0)	

*VOR, voriconazole; FZ, fluconazole; PZ, posaconazole; IT, itraconazole; NWT, non-wild type; WT, wild type.*

## Discussion

During 2012–2018, a total of 2,048 *Candida* isolates were identified from positive blood cultures. *C. tropicalis* was ranked as the most common cause of candidemia (41.1–48.7%; mean 46.7%; Supplementary Figure S2). The prevalence of *C. tropicalis* causing candidemia among candidemia patients at Siriraj Hospital has increased considerably from 28.0% during 2006–2009 to 46.7% during 2012–2018 (Boonyasiri et al., 2013). A study reported that the prevalence in Thailand was almost 50% during 2010–2011 in Thailand (Tan et al., 2015). Compared with the average for other Asian countries (25.4%), the proportion of *C. tropicalis* among candidemia patients in Thailand was much higher. In fact, a large surveillance study of candidemia in Asia indicated that the proportion of *C. tropicalis* was higher in tropical regions (46.2%), such as India, Singapore, and Thailand, than that for temperate regions (18.9%) (Tan et al., 2015). Furthermore, our study demonstrated that *C. tropicalis* was the predominant cause of candidemia, followed by *C. albicans*. This shift in species distribution of invasive candidiasis has also been found in India and Pakistan (Farooqi et al., 2013; Chakrabarti et al., 2015).

The frequency of infections by *C. tropicalis* is increasing (Guinea, 2014). One study has reported that the disease characteristics and prior antifungal treatment are involved in the change in the prevalence of the non-albicans *Candida* (Makanjuola et al., 2018). Therefore, *in vitro* virulence and antifungal susceptibility studies of the *C. tropicalis* blood isolates were performed.

Previous findings have suggested that the ability to produce the two hydrolytic enzymes, to secrete the hemolytic enzyme, and to form the biofilm are key virulence factors that facilitate hematogenous infections by *Candida* spp. (Gokce et al., 2007; Deorukhkar et al., 2014). Although the secretion of phospholipase is considered a key contribution of host invasion, none of our studied isolates produced the enzyme. Our finding was similar to previous analysis in *Candida* spp. (Samaranayake et al., 1984) which reported that *C. tropicalis* has no ability to secrete phospholipase. Although another study reported a high percentage of *C. tropicalis* isolates secreted phospholipase, the isolates exhibited very low production (Galan-Ladero et al., 2010; Jiang et al., 2016). On the other hand, among non-albicans *Candida* spp., the highest production of phospholipase was found in *C. tropicalis* in another report (Deorukhkar et al., 2014). However, this might not be applicable in Thailand as a previous study showed more than 90% of the *C. parapsilosis* sensu stricto isolates produced phospholipase (Pharkjaksu et al., 2018). The variability of results might be brought by biological differences among isolates and sensitivity of phospholipase detection methods. In addition, previous exposure of antifungal drugs such as nystatin and amphotericin B causes a significant reduction of phospholipase activity (Anil and Samaranayake, 2003). Unfortunately, information on prior drug treatment was not evaluated in the present study.

Typically, the fluconazole-resistant rates of *C. tropicalis* only range from 1.1 to 2.5% in the United States and Europe. Although as high as a 10% triazole resistance rate has been reported in the Asia-Pacific region (Pfaller et al., 2019), the resistance of *C. tropicalis* was much higher in this study with one-fifth of isolates being resistant to voriconazole and fluconazole, and high MICs to posaconazole and itraconazole. Comparing to a previous study in Thailand during 1999–2002, the fluconazole-resistant rate of *C. tropicalis* substantially increased from 0 to 43.8% in this study (Foongladda et al., 2004). In contrast, a recent study reported a very low resistance/non-wild type rate of *C. albicans* to all antifungal agents (Pham et al., 2019). Because most antibiotics, including all the triazoles, has been available over the counter in Thailand as both topical and oral form for at least the past 40 years (Aswapokee et al., 1990; Hoge et al., 1998), the susceptible *C. albicans* in the skin and mucosal microbiota might be “selected out” by the overuse of triazoles. Finally, with the higher frequency of *C. tropicalis* isolates in skin and microbiota, the higher *C. tropicalis* candidemia is to be expected. However, this remains to be proven by further microbiota screening among the Thai population.

Multilocus sequence typing and phylogenetic analysis of the *C. tropicalis* isolates in this study showed high genetic diversity, with as many as 22 DSTs being identified from the 48 isolates in this study. As expected, an eBURST group associated with the four triazole resistance was found. As a previous study in Taiwan also reported a cluster associated with fluconazole resistance in Taiwan (Chou et al., 2007), we investigated if this would also be the case in Thailand. Interestingly, this eBURST group of the four azoles resistant isolates also included the resistant isolates from Taiwan and China identified previously (Chou et al., 2007; Fan et al., 2017). This implied that this eBURST group 3 is strongly associated with the triazole resistance of Asian *C. tropicalis* isolates.

Finally, the ability to express *in vitro* virulence and antifungal susceptibility pattern between *C. albicans* and *C. tropicalis* were compared. Information on *in vitro* virulence and antifungal susceptibility of 46 *C. albicans* blood isolates was retrieved from our recent study (Pham et al., 2019). By comparing to the *C. albicans* blood isolates which isolated at the same time, the *C. tropicalis* exhibited significantly more overall proteinase activity and stronger hemolytic activity. Moreover, the *C. tropicalis* blood isolates showed higher resistance to voriconazole and fluconazole, and higher MICs to posaconazole (Supplementary Tables S1, S2). In fact, a previous study of *Candida* spp. in Thailand in 1999–2002 reported that all *C. tropicalis* isolates were susceptible to fluconazole (Foongladda et al., 2004). This suggests the antifungal resistance of the *C. tropicalis* blood isolates did occur only recently. Unfortunately, the *in vitro* virulence has never been studied in Thailand before this study. However, the high hemolytic activity was reported in *C. tropicalis* blood isolates previously (Favero et al., 2014; Seneviratne et al., 2016) and the predominant MLST clade 17 of *C. albicans* blood isolates showed significant stronger hemolytic activity than the less common clade (Pham et al., 2019). These suggest *in vitro* virulence could somewhat influence the pathogenesis of the yeasts. Taken together, the higher *in vitro* virulence and antifungal drug resistance were collectively responsible for the prevalence shift of *C. tropicalis* among candidemia patients.

## Conclusion

An increased prevalence of *C. tropicalis* among candidemia patients during the past 10 years has been reported. The azole resistance/high MICs isolates are strongly associated with the eBURST group 3 based on the MLST analysis. Finally, *C. tropicali*s blood isolates exhibited higher proteinase activity, hemolytic activity, and antifungal drug resistance/high MIC rates than the *C. albicans* blood isolates. These implied that these virulence phenotypes and antifungal resistance were collectively responsible for the prevalence shift of *C. tropicalis*. However, although the *post hoc* analysis confirmed sufficient isolates for the association analysis, the 48 isolates might not perfectly represent the molecular distribution of *C. tropicalis* in Thailand. Therefore, a further study with more isolates per time point is needed.

## Data Availability Statement

All datasets generated for this study are included in the article/Supplementary Material.

## Ethics Statement

Upon approval by the Siriraj Institutional Review Board (COA number: SI 091/2016), information on all Candida isolates from positive hemocultures during 2012-2018 were collected from the Department of Microbiology, Faculty of Medicine Siriraj Hospital, Mahidol University, Bangkok, Thailand.

## Author Contributions

PN and PC designed the study. OT and SP performed the experiments and analyzed the data. OT and PN wrote the manuscript. All authors read the manuscript.

## Conflict of Interest

The authors declare that the research was conducted in the absence of any commercial or financial relationships that could be construed as a potential conflict of interest.

## Footnotes

1 http://pubmlst.org/ctropicalis, accessed in August 2019

2 http://www.megasoftware.net/

3 http://pubmlst.org/ctropicalis/

4 http://www.phyloviz.net/goeburst/

5 https://www.icalcu.com/stat/chisqtest.html

## Supplementary Material

FIGURE S1 Minimum spanning tree illustrating relationships between the 48 *C. tropicalis* isolates from Thailand and 1,019 isolates from other countries available from the *C. tropicalis* MLST database as of August 2019. Each circle corresponds to a unique DST; the number outside the circle indicates an eBURST cluster; the size of the circle represents the number of isolates belonging to the same DST; and the colors inside the circle represent the origin country of the *C. tropicalis* isolates.

FIGURE S2 Species distribution of *Candida* species isolated from positive blood cultures at Siriraj Hospital during 2012–2018.

TABLE S1 *In vitro* virulence comparison between *C. tropicalis* and *C. albicans* blood isolates.

TABLE S2 Antifungal susceptibility comparison between *C. tropicalis* and *C. albicans* blood isolates.
