# Supplementary figures and images for: An Association of an eBURST Group With Triazole Resistance of Candida tropicalis Blood Isolates
Source: Front Microbiol. 2020 May 19;11:934. doi: 10.3389/fmicb.2020.00934 (PMC7248567; doi:10.3389/fmicb.2020.00934)

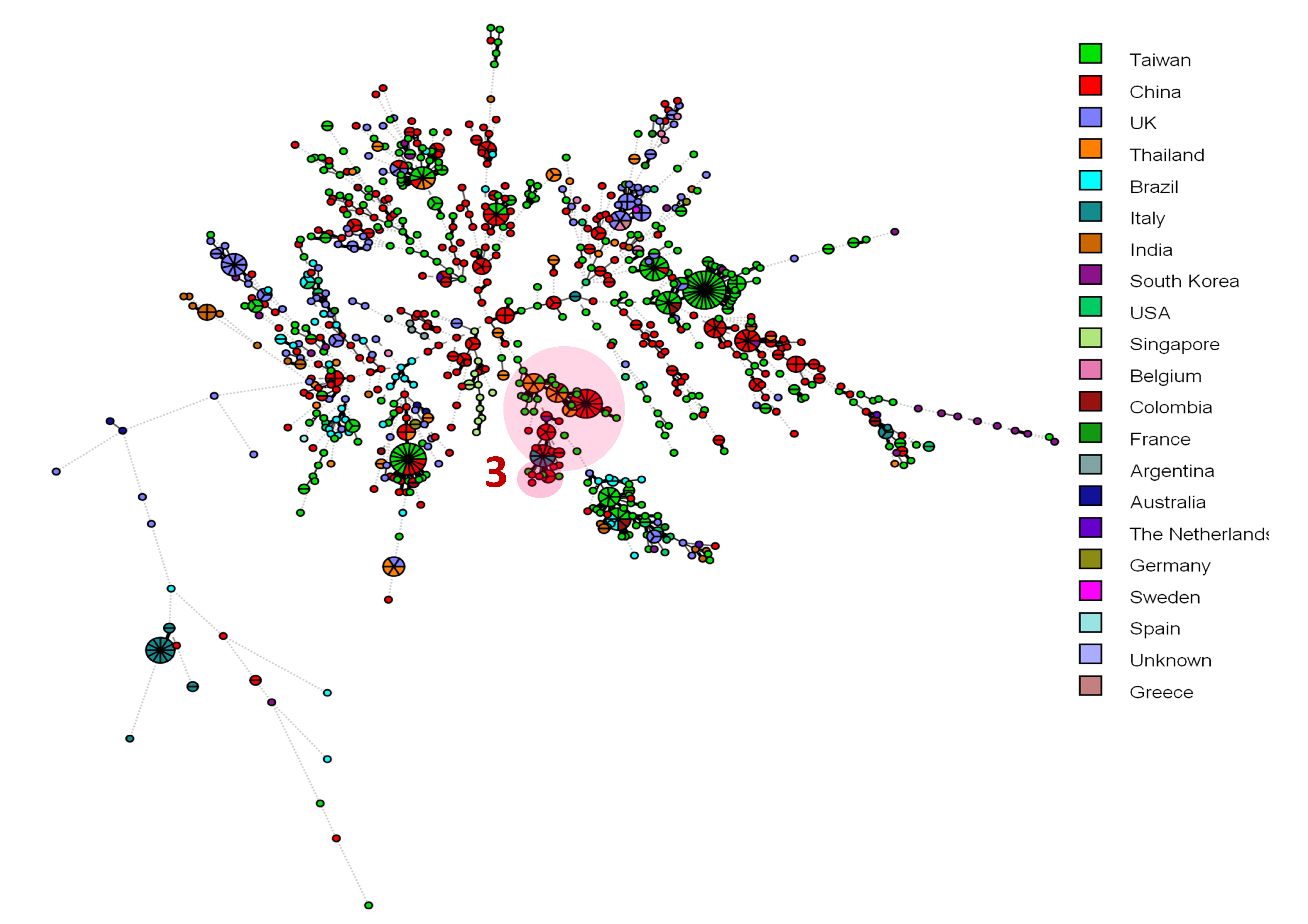

Supplement: FIGURE S1 — Minimum spanning tree illustrating relationships between the 48 C. tropicalis isolates from Thailand and 1,019 isolates from other countries available from the C. tropicalis MLST database as of August 2019. Each circle corresponds to a unique DST; the number outside the circle indicates an eBURST cluster; the size of the circle represents the number of isolates belonging to the same DST; and the colors inside the circle represent the origin country of the C. tropicalis isolates. [file Image_1.jpeg]

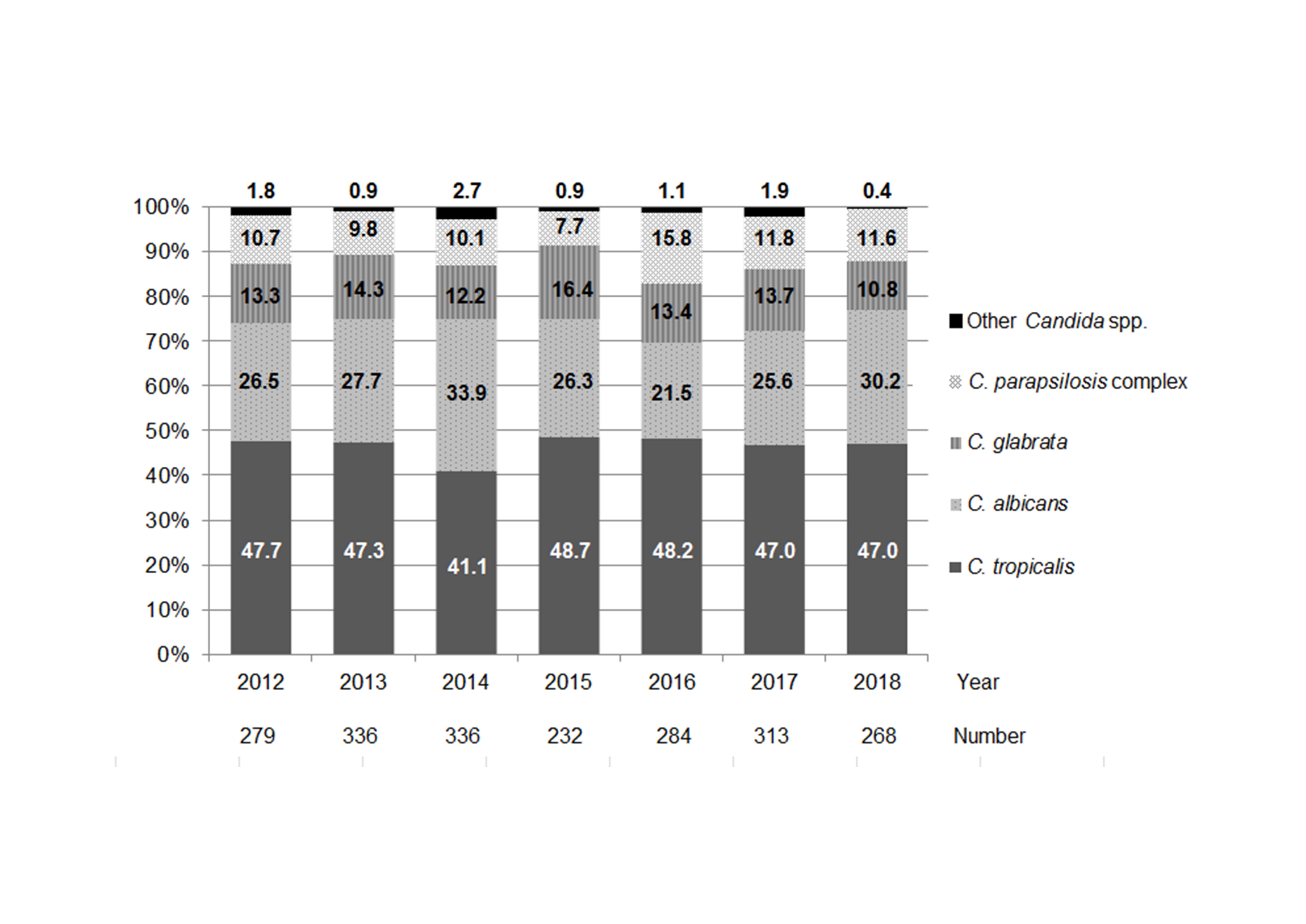

Supplement: FIGURE S2 — Species distribution of Candida species isolated from positive blood cultures at Siriraj Hospital during 2012–2018. [file Image_2.jpeg]
