# Supplementary material for: An Association of an eBURST Group With Triazole Resistance of Candida tropicalis Blood Isolates
Source: Front Microbiol. 2020 May 19;11:934. doi: 10.3389/fmicb.2020.00934 (PMC7248567; doi:10.3389/fmicb.2020.00934)
Supplement: TABLE S1 — In vitro virulence comparison between C. tropicalis and C. albicans blood isolates. [file Table_1.pdf]

Supplementary Table S1: *In vitro* virulence comparison of *C. tropicalis* and *C. albicans* blood isolates

Virulent factors	Species	Number (%) of isolates					P-value
		Negative	Weak/ Low	Medium/ Positive	Strong/ Strong positive/ High	Very strong	
Proteinase activity[a]	*C. tropicalis*	34 (70.8)	0 (0)	3 (6.3)	11 (22.9)	0 (0)	< 0.001
	C. albicans	46 (100)	0 (0)	0 (0)	0 (0)	0 (0)	
Phospholipase activity[a]	*C. tropicalis*	48 (100)	0 (0)	0 (0)	0 (0)	0 (0)	< 0.001
	C. albicans	0 (0)	0 (0)	3 (5.5)	13 (28.3)	30 (65.2)	
Hemolytic activity[b]	*C. tropicalis*	0 (0)	NA	10 (20.8)	38 (79.2)	NA	< 0.001
	C. albicans	0 (0)	NA	28 (60.9)	18 (39.1)	NA	
Biofilm formation[c]	*C. tropicalis*	3 (6.2)	19 (39.6)	NA	26 (54.2)	NA	0.185
	C. albicans	8 (17.4)	13 (28.3)	NA	25 (54.3)	NA	

Notes: [a]Proteinase and phospholipase activity: negative, weak, medium, strong, very strong; [b]Hemolytic activity: negative, positive, strong positive; [c]Biofilm formation: negative, low, high; NA: not applicable
