# Supplementary material for: An Association of an eBURST Group With Triazole Resistance of Candida tropicalis Blood Isolates
Source: Front Microbiol. 2020 May 19;11:934. doi: 10.3389/fmicb.2020.00934 (PMC7248567; doi:10.3389/fmicb.2020.00934)
Supplement: TABLE S2 — Antifungal susceptibility comparison between C. tropicalis and C. albicans blood isolates. [file Table_2.pdf]

Supplementary Table S2: Antifungal susceptibility comparison between *C. tropicalis* and *C. albicans* blood isolates

Antifungal drugs	Species	Category number (%)			P-value
		S/WT	SDD/I	R/NWT	
Voriconazole	*C. tropicalis*	21 (43.8)	6 (12.4)	21 (43.8)	< 0.001
	C. albicans	45 (97.8)	0 (0)	1 (2.2)	
Fluconazole	*C. tropicalis*	26 (54.1)	1 (2.1)	21 (43.8)	< 0.001
	C. albicans	45 (97.8)	0 (0)	1 (2.2)	
Posaconazole[a]	*C. tropicalis*	30 (62.5)	NA	18 (37.5)	< 0.001
	C. albicans	45 (97.8)	NA	1 (2.2)	
Anidulafungin	*C. tropicalis*	47 (97.9)	0 (0)	1 (2.1)	0.325
	C. albicans	46 (100)	0 (0)	0 (0)	
Micafungin	*C. tropicalis*	47 (97.9)	0 (0)	1 (2.1)	0.325
	C. albicans	46 (100)	0 (0)	0 (0)	
Caspofungin	*C. tropicalis*	47 (97.9)	0 (0)	1 (2.1)	0.325
	C. albicans	46 (100)	0 (0)	0 (0)	
Amphotericin B[a]	*C. tropicalis*	48 (100)	NA	0 (0)	NA
	C. albicans	46 (100)	NA	0 (0)	

Notes: [a]categorization based on the epidemiologic cut-off values (ECVs); Itraconazole and 5-flucytosine interpretation were not available for *C. albicans* Abbreviations: MIC, minimal inhibitory concentration; GM, geometric mean; S, susceptible; WT, wild type; SDD, susceptible-dose dependent; I, intermediate; R, resistant; NWT, non-wild type; NA, not applicable
